# Berberine Suppresses Adipocyte Differentiation via Decreasing CREB Transcriptional Activity

**DOI:** 10.1371/journal.pone.0125667

**Published:** 2015-04-30

**Authors:** Juan Zhang, Hongju Tang, Ruyuan Deng, Ning Wang, Yuqing Zhang, Yao Wang, Yun Liu, Fengying Li, Xiao Wang, Libin Zhou

**Affiliations:** Shanghai Clinical Center for Endocrine and Metabolic Diseases, Shanghai Institute of Endocrine and Metabolic Diseases, Department of Endocrine and Metabolic Diseases, Ruijin Hospital, Shanghai Jiaotong University School of Medicine, Shanghai, China; National University of Singapore, SINGAPORE

## Abstract

Berberine, one of the major constituents of Chinese herb Rhizoma coptidis, has been demonstrated to lower blood glucose, blood lipid, and body weight in patients with type 2 diabetes mellitus. The anti-obesity effect of berberine has been attributed to its anti-adipogenic activity. However, the underlying molecular mechanism remains largely unknown. In the present study, we found that berberine significantly suppressed the expressions of CCAAT/enhancer-binding protein (C/EBP)α, peroxisome proliferators-activated receptor γ2 (PPARγ2), and other adipogenic genes in the process of adipogenesis. Berberine decreased cAMP-response element-binding protein (CREB) phosphorylation and C/EBPβ expression at the early stage of 3T3-L1 preadipocyte differentiation. In addition, CREB phosphorylation and C/EBPβ expression induced by 3-isobutyl-1-methylxanthine (IBMX) and forskolin were also attenuated by berberine. The binding activities of cAMP responsive element (CRE) stimulated by IBMX and forskolin were inhibited by berberine. The binding of phosphorylated CREB to the promoter of C/EBPβ was abrogated by berberine after the induction of preadipocyte differentiation. These results suggest that berberine blocks adipogenesis mainly via suppressing CREB activity, which leads to a decrease in C/EBPβ-triggered transcriptional cascades.

## Introduction

Obesity has become a major world public health problem. It is associated with increased risks of many diseases such as type 2 diabetes, hypertension, coronary heart disease, fatty liver, and osteoarthritis [1]. The expansion of adipose tissue results not only from increased adipocyte size (hypertrophy), but also from increased adipocyte numbers (hyperplasia). Hyperplasia occurs via *de novo* differentiation of preadipocytes, which are located in the stromal-vascular fraction of adipose tissue [2]. Thus, the regulation of adipocyte differentiation is crucial for the prevention and treatment of obesity and its related diseases.

3T3-L1 cell line derived from 3T3 Swiss mouse embryo is one of the most well-characterized and reliable models for studying adipogenesis. Confluent 3T3-L1 preadipocytes differentiate upon exposure to the adipogenic inducers such as insulin, 3-isobutyl-1-methylxanthine (IBMX), and dexamethasone [3]. The development of fully differentiated mature adipocytes from precursor cells is an elegant progression involving the sequential activation of a battery of transcription factors [4]. Elevation of cellular cAMP concentration has been associated with crucial events in the early program of adipocyte differentiation, involving in cAMP-responsive element-binding protein (CREB) activation. Activated CREB induces expression of CCAAT/enhancer-binding protein (C/EBP)β, triggering expression of a number of transcription factors, including C/EBPα and peroxisome proliferators-activated receptor γ2 (PPARγ2) [5].

Natural compounds that inhibit adipogenic differentiation are frequently screened to develop the anti-obesity drugs. Berberine, one of the major constituents of Chinese herb Rhizoma coptidis, is an isoquinoline alkaloid. It has been demonstrated that berberine treatment reduces body weight, blood glucose and lipid levels in clinical and experimental studies [6–8]. Previous studies showed that berberine inhibited the differentiation of 3T3-L1 preadipocytes via decreasing PPARγ2 expression [9–11]. But the molecular mechanism remains largely unknown. AMP-activated protein kinase (AMPK) is emerging as a metabolic master switch. Berberine has been shown to activate AMPK, which contributes to the beneficial metabolic effects of berberine in peripheral tissues [7,12–14]. However, we previously found that berberine decreased insulin secretion and lipolysis via cAMP/ protein kinase A (PKA) pathway independent of AMPK activation in pancreatic β-cells and adipocytes [8,15]. Therefore, it is possible that berberine suppresses adipocyte differentiation via down-regulation of cAMP dependent pathway.

In the present study, we found that berberine significantly inhibited the differentiation of 3T3-L1 preadipocytes, CREB phosphorylation, and C/EBPβ expression. In addition, berberine prevented the binding of phosphorylated CREB to the promoter of C/EBPβ. Therefore, our results suggest that berberine suppresses adipogenesis via inhibiting the activities of CREB and C/EBPβ, which leads to decreased expressions of C/EBPα and PPARγ2 as well as other adipogenic genes.

## Materials and Methods

### Materials

Dulbecco's modified Eagle's medium (DMEM) and other culture reagents were obtained from Gibco Life Technologies (Grand Island, NY).The cell culture plates were purchased from Nalge Nunc International (Roskilde, Denmark). Human insulin (HumulinR) was from Eli Lilly S.A.S.(Fegersheim, France). Bovine serum albumin (BSA), forskolin, IBMX, and dexamethasone were purchased from Sigma (St Louis, MO, USA). Compound C was purchased from Calbiochem (San Diego, CA). Anti-CREB, anti-phospho-CREB (Ser133), anti-PPARγ, anti-C/EBPα, anti-fatty acid synthase (FAS), anti-fatty acid binding protein 4 (FABP4), anti-C/EBPβ, anti-AMPK, anti-phospho-AMPK (Thr172), anti-acetyl-CoA carboxylase (ACC), anti-phospho-ACC(Ser79), anti-β-actin, anti-α1-tubulin, anti-mouse IgG and anti-rabbit IgG conjugated with horseradish peroxidase were from Cell Signaling Technology (Beverly, MA, USA). Murine-derived 3T3-L1 preadipocytes were purchased from American Type Culture Collection (Rockville, MD). Berberine was obtained from the National Institute for the Control of Pharmaceutical and Biological Products (Beijing, China).

### 3T3-L1 cell culture and differentiation

3T3-L1 preadipocytes were grown and passaged in DMEM containing 25 mM glucose plus 10% fetal bovine serum (FBS). For adipocyte differentiation, 2-day postconfluent cells were placed in 10% FBS-DMEM with 0.5 mM IBMX, 0.25 μM dexamethasone, and 1 μg/ml insulin (designated hereafter as MDI). After 2 days, the medium was changed to 10% FBS-DMEM containing 1 μg/ml insulin alone for 2 additional days and was replaced with 10% FBS-DMEM. Thereafter, the medium was changed every 2 days.

### Oil red O staining

3T3-L1 adipocytes induced for various days were washed with PBS, fixed with 4% paraformaldehyde in 0.1 M phosphate buffer, pH 7.4 for 15 min at room temperature, and washed three times with deionized water. A mixture of oil red-O (0.6% oil red-O dye in isopropanol) and water at a 6:4 ratio was layered on the cells for 10 min. The cells were then rinsed four times with PBS before visualization and documentation. Following the microscopic observation, an extraction solution (4% NP40) was added to cells and the absorbance of the extracted dye was spectrophotometrically measured at 490 nm using a microplate reader (PowerVave XS2, BIOTEK,America).

### Real-time RT-PCR

Total RNA was isolated with Trizol reagent (Invitrogen) and reverse transcribed from Random Primers (Promega) according to the manufacturer’s instructions. Real-time PCR was performed by Roche LightCycler 480 system using SYBR Premix Ex Taq (Takara, Japan) in final volume of 10 μl. The conditions of real-time PCR were conducted as follows: denaturation at 95°C for 10 s, 40 cycles at 95°C for 5 s, 60°C for 31 s. A melting curve was built in the temperature range of 60–95°C at the end of the amplification. All primers were synthesized by Shanghai Biological Engineering Technology & Services Co., Ltd. 18S was amplified as an internal control. The primer sequences used for real-time PCR were designed as follows: 18S forward primer 5’- ACCGCAGCTAGGAATAATGGA-3’ and reverse primer 5’- GCCTCAGTTCCGAAAACCA-3’; C/EBPα forward primer 5’-CAAGAACAGCAACGAGTACCG-3’ and reverse primer 5’-GTCACTGGTCAACTCCAGCAC-3’; PPARγ2 forward primer 5’-GCATGGTGCCTTCGCTGA-3’ and reverse primer 5’-TGGCATCTCTGTGTCAACCATG-3’; sterol regulatory element-binding protein 1c (SREBP1c) forward primer 5’- ACGGAGCCATGGATTGCAC-3’ and reverse primer 5’-TGTCTCACCCCCAGCATAG-3’; lipoprotein lipase (LPL) forward primer 5’-ATGGATGGACGGTAACGGGAA-3’ and reverse primer 5’-CCCGATACAACCAGTCTACTACA-3’.

### Western blotting

Cells in six-well plates were washed twice with ice-cold PBS and placed immediately in lysis buffer containing 1 mM phenylmethylsulfonyl fluoride (PMSF), protease inhibitor cocktail I (Calbiochem), and phosphatse inhibitor cocktail V (Merk). Lysates were gently mixed for 10 min at 4°C and then centrifuged at 13,000×g for 15 min at 4°C. The protein concentration of the extracts was determined according to the method of Bradford, using BSA as the standard. Samples were separated by SDS-PAGE and transferred to PVDF-Plus membranes (Bio-Rad, Hercules, CA). The transferred membranes were blocked, washed, and incubated with various primary antibodies, followed by horseradish peroxidase-conjugated secondary antibody. Blotted membrane was developed with ECL Advance (Cell Signaling Technology, Boston, MA) and imaged with a LAS-4000 Super CCD Remote Control Science Imaging System (Fuji, JAP).

### Luciferase reporter assay

After cells were plated in 24-well plates for 24 h, each well of cells was transfected with 800 ng of cyclic AMP response element (CRE) luciferase reporter plasmid by lipofectamine2000 (Invitrogen) according to the manufacturer’s instructions. 24 h later, the cells were treated with forskolin or IBMX in the presence or absence of berberine for another 6 h (293T cells) or 12 h (3T3-L1 preadipocytes). pRL-SV40 expressing renilla luciferase (Promega) was used to normalize the luciferase activity. Cells were harvested and luciferase activity was measured using the Dual-Luciferase Reporter Assay System (Promega).

### Chromatin immunoprecipitation analysis

Chromatin immunoprecipitation (ChIP) analyses were performed using an assay kit (Millipore, USA) according to the manufacturer’s protocol. Two-day post-confluent 3T3-L1 cells were treated with MDI in the present or absence of berberine for 24 h, after which cells were cross-linked with 1% formaldehyde for 10 min at room temperature, and quenched with 1ml 10 × glycine. After washing twice with ice-cold PBS containing protease inhibitors, scraping and centrifugation, cell pellets were resuspended in SDS lysis buffer. After incubation for 10 min at 4°C, the cell lysates were sonicated 12 times with each time being 10s using a sonicator (soniprep150, Fisher Scientific). After centrifugation, the supernatant was diluted in ChIP dilution buffer and then incubated overnight at 4°C with anti-phospho-CREB (Cell signaling). Immune complexes were recovered by the addition of 60 μl salmon sperm DNA/protein A-agarose-50% slurry and incubation for 2 h at 4°C with rotation. Agarose beads were pelleted by gentle centrifugation (1000×g at 4°C).The beads were sequentially washed with low and high salt buffer, LiCl buffer, and finally twice with TE (Tris, 10 mM, pH 8.0; EDTA, 1 mM) buffer. After washing, the immune complexes were eluted by incubation for 15 min at 25°C with 200 μl fresh elution buffer (1% SDS, 0.1 M NaHCO3). To reverse the cross-linking of DNA, 8 μl of 5 M NaCl was added and incubated overnight at 65°C. After treatment with RnaseA for 1 h and proteinase K for 2 h at 45°C, DNA was recovered by phenol-chloroform extraction and ethanol precipitation. The DNA pellets were resuspended in 50 μl TE buffer. PCR amplification was carried out for 35 cycles with 2 μl sample DNA solution, and PCR products were separated on 2% agarose gels in 1×TBE. The primers were used to amplify the segment (-121 to +31) flanking the two CRE-binding sites of C/EBPβ with forward primer 5’-GGCCGGGCAATGACGCGCAC-3’ and reverse primer 5‘-GGCTCCGCTGCGTCCCGGTCC-3’.

### Statistics

Data are presented as mean ± SD. Significance between groups was determined using an unpaired two-tailed Student's t-test or one-way ANOVA when appropriate. Significance was established at *P*<0.05.

## Results

### Anti-adipogenic effect of berberine

**Fig 1:**
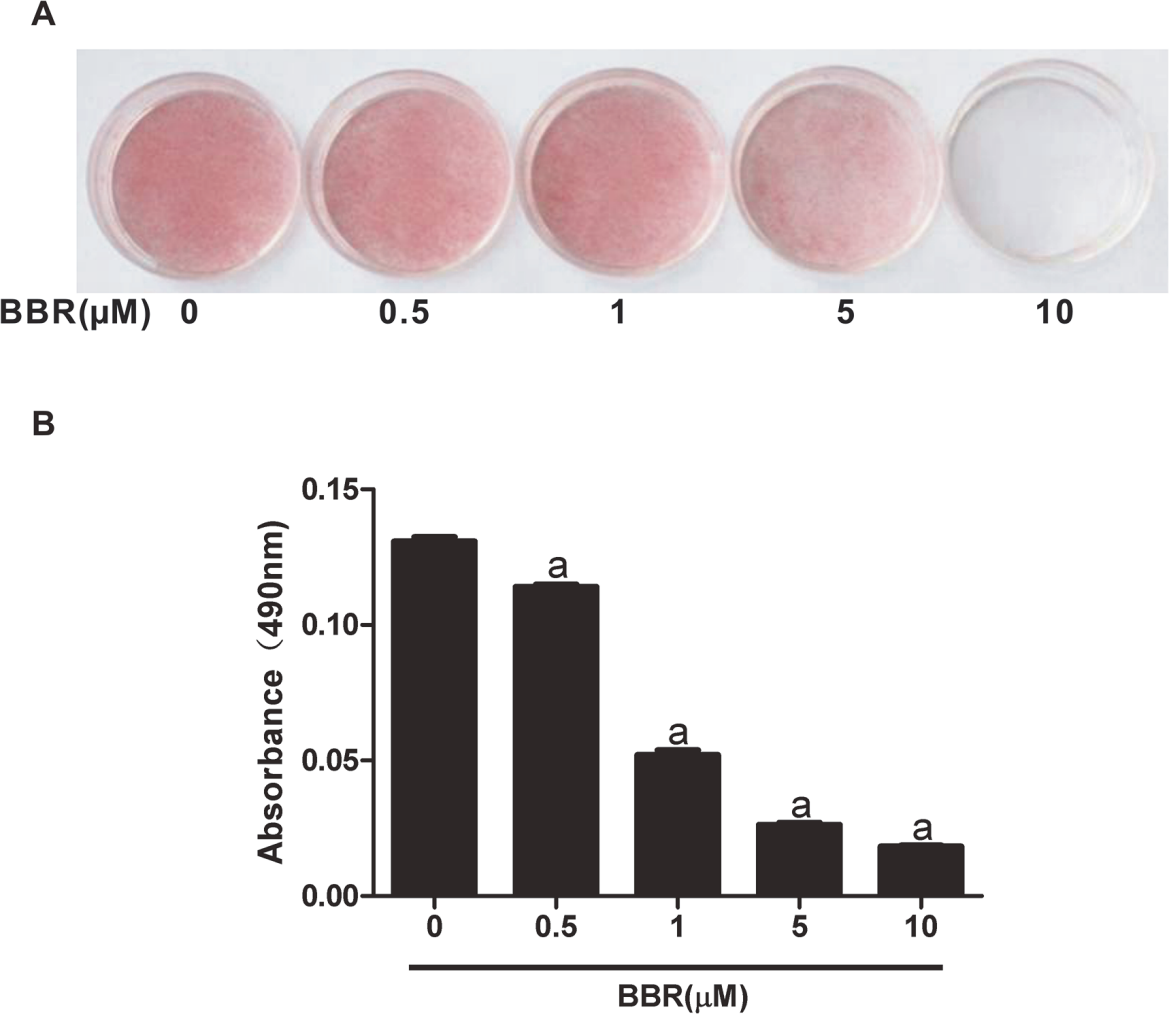
Berberine inhibits the differentiation of 3T3-L1 preadipocytes. (A) 3T3-L1 cells were induced to differentiate in the presence of various concentrations of berberine (BBR). 7 days later, the cells were stained with oil red O and photographed. (B) Oil red O staining was quantitatively analyzed. Data are presented as mean ± SD (*n* = 4). ^a^ *P*<0.01 compared with 0 μM BBR.

**Fig 2:**
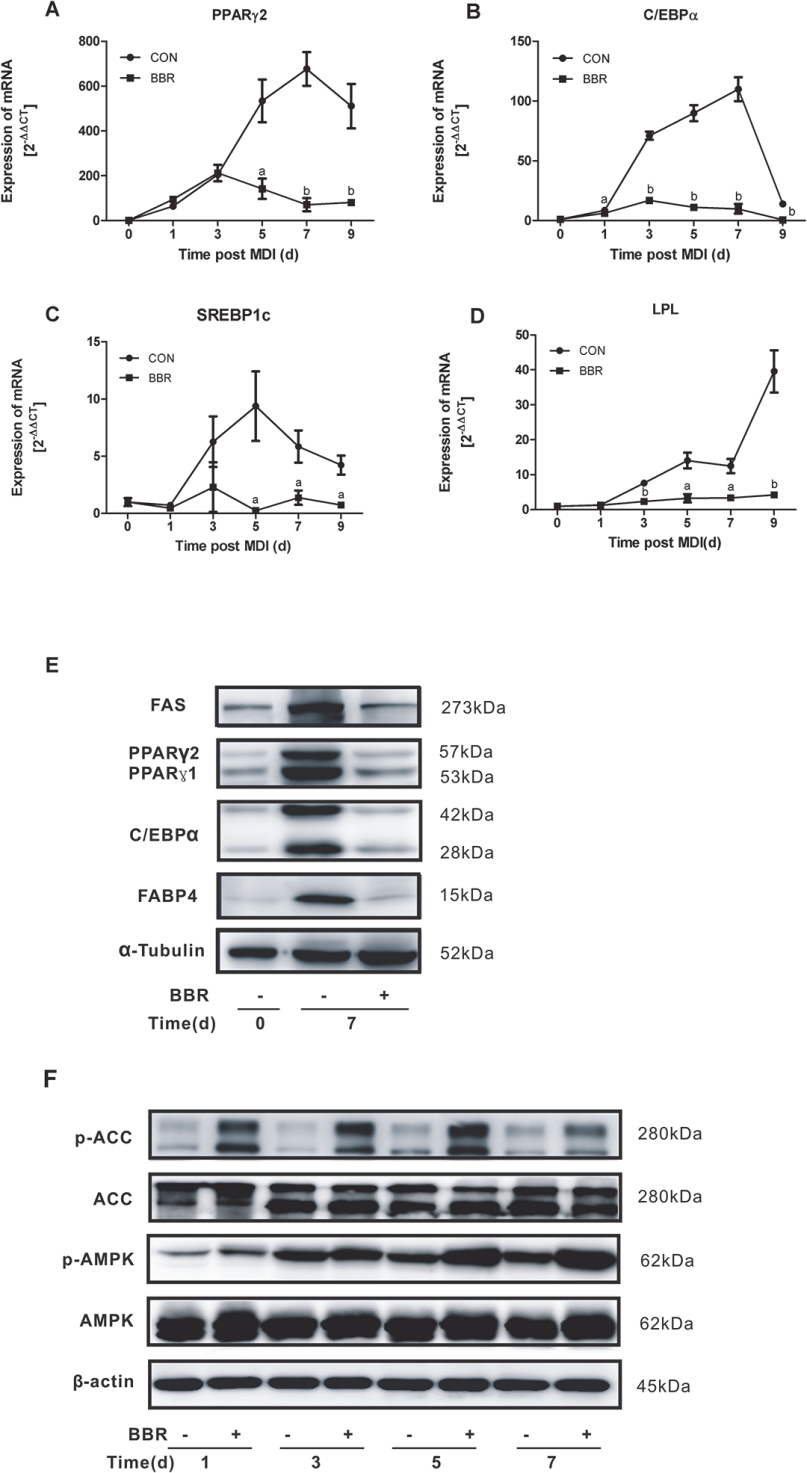
Effects of berberine on the expressions of adipogenic genes and AMPK activity during 3T3-L1 adipocyte differentiation. 3T3-L1 preadipocytes were induced to differentiate for various days in the presence or absence of 5 μM berberine (BBR). Peroxisome proliferators-activated receptor (PPAR)γ2, CCAAT/enhancer-binding protein (C/EBP)α, sterol regulatory element-binding proteins (SREBP)1c, lipoprotein lipase (LPL) mRNA expressions were detected by real-time PCR (A-D). The protein expressions of C/EBPα, PPARγ, fatty acid synthase (FAS), fatty acid binding protein 4 (FABP4) were detected by Western blot (E). The phosphorylations of AMPK and ACC were detected by Western blot (F). Data are represented as mean ± SD. ^a^ *P*<0.05 and ^b^ *P*<0.01 compared with control. A representative blot from three independent experiments is shown. All three experiments showed similar results.

In the present study, we firstly determined the dose-dependent effect of berberine on adipocyte differentiation. As shown in Fig 1A and 1B, a large amount of lipid was accumulated in mature 3T3-L1 adipocytes after the induction of differentiation for 7 days. When berberine was added to the culture medium throughout the process of differentiation, 3T3-L1 adipocyte differentiation was significantly inhibited in a dose-dependent manner, with an obvious effect at the concentration of 5 μM. We further examined the time-dependent effect of berberine on adipogenic gene expressions by real-time PCR. With the maturity of 3T3-L1 adipocyte differentiation, the mRNA expressions of C/EBPα, PPARγ2, SREBP1c, and LPL increased gradually. However, the increased expressions of these genes were suppressed in the presence of 5 μM berberine (Fig 2A–2D). On day 7 of adipocyte differentiation, the protein expressions of four adipogenic marker genes including C/EBPα, PPARγ, FAS, and FABP4 were strongly induced, which were abrogated by berberine (Fig 2E).

It has been demonstrated that AMPK activation inhibits adipocyte differentiation [16–19]. In many cells and tissues, berberine activates AMPK [7, 8, 14, 15]. As expected, our study showed that berberine stimulated the phosphorylations of AMPK and its substrate ACC over the entire time course of 3T3-L1 adipocyte differentiation (Fig 2F). However, our previous studies revealed that berberine stimulated glucose transport and suppressed lipolysis and insulin secretion independent of AMPK activation [8, 15, 20]. In the current study, we found that compound C, a specific AMPK inhibitor, blocked 3T3-L1 adipocyte differentiation (data not shown), which is in consistent with a previous study [21]. Therefore, we supposed that berberine could inhibit adipogenesis via pathways other than AMPK activation.

### Berberine suppresses the initiation of adipocyte differentiation

**Fig 3:**
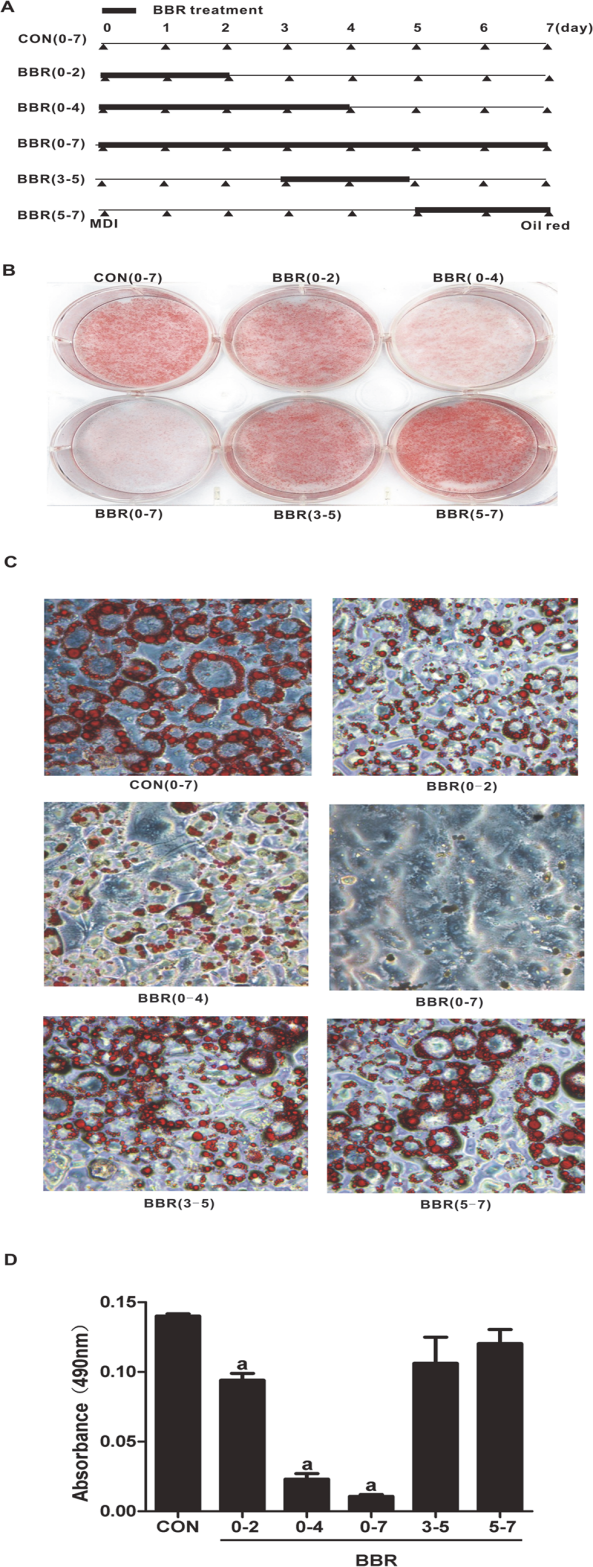
Berberine suppresses the initiation of adipocyte differentiation. (A) 3T3-L1 preadipocytes were incubated with 5 μM berberine (BBR) for the indicated time periods during the MDI-induced differentiation program. Thick lines indicate berberine treatment. (B and C) 3T3-L1 cells induced to differentiate for 7 days were fixed in 4% paraformaldehyde and stained with oil red O. (D) Oil red O staining was quantitatively analyzed. Data are presented as mean ± SD (*n* = 4). ^a^ *P*<0.01 compared with control (CON).

To study the time course of the inhibitory effect of berberine on adipocyte differentiation, 3T3-L1 preadipocytes were treated with 5 μM berberine at different stages of the differentiation (Fig 3A) and stained by oil red O on day 7. As shown in Fig 3B–3D, berberine treatment during day 0–2, day 0–4, and day 0–7 resulted in significant inhibition of intracellular lipid accumulation while 3T3-L1 adipocytes exposed to berberine on day 3–5 and day 5–7 were able to fully differentiate into mature adipocytes. These results indicate that berberine exerts a profoundly inhibitory effect at the early stage of preadipocyte differentiation.

### Berberine inhibits CREB activation and C/EBPβ expression during adipocyte differentiation

**Fig 4:**
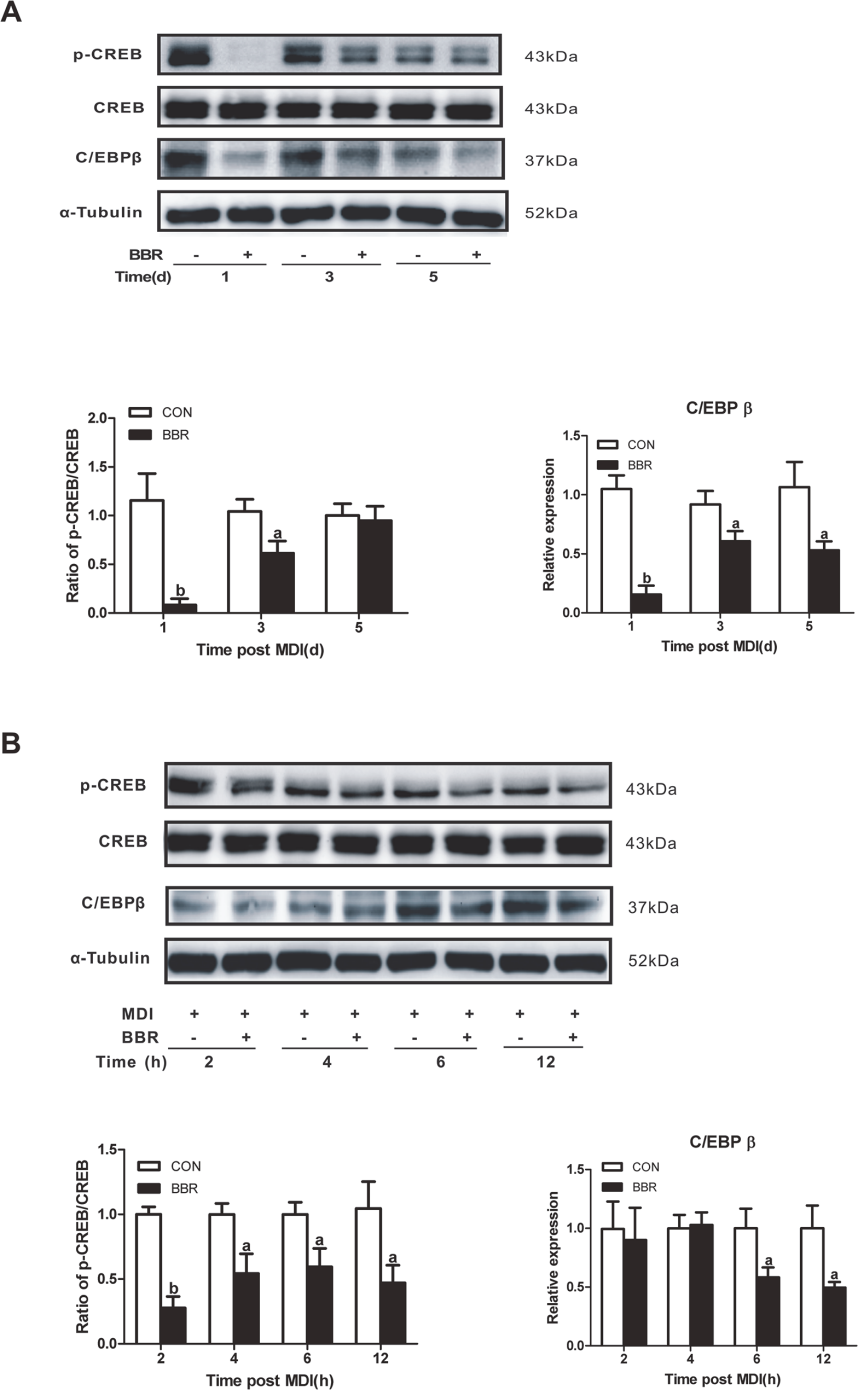
Inhibition of CREB phosphorylation and C/EBPβ expression by berberine during the process of differentiation. (A) 3T3-L1 preadipocytes were induced to differentiate according to the standard protocol for 5 days in the presence or absence of 5 μM berberine (BBR). (B) 3T3-L1 preadipocytes were stimulated with MDI for the indicated time in the presence or absence of 5 μM berberine. CREB phosphorylation and C/EBPβ expression was assayed by Western blot. The scanned bar graphs show relative folds of phosphorylated CREB over CREB or C/EBPβ protein expression. Data are represented as mean ± SD (*n* = 3). ^a^ *P*<0.05 and ^b^ *P*<0.01 compared with control (CON).

It is well known that CREB is crucial for initiating the adipocyte differentiation process [22]. Therefore, we postulated that CREB could be a critical target for the anti-adipogentic effect of berberine. As expected, berberine markedly reduced CREB phosphorylation on day 1 and day 3 of 3T3-L1 preadipocyte differentiation (Fig 4A). We further observed the short-term effect of berberine on CREB activity and found that berberine decreased CREB phosphorylation 2 hours after MDI stimulation (Fig 4B).

Adipogenesis is orchestrated by the expression of master adipogenic regulators [23]. As a target gene of CREB, C/EBPβ is an early regulator of preadipocyte differentiation. C/EBPβ transcriptionally activates two genes encoding for major adipogenic transcription factors, C/EBPα and PPARγ2 [22]. In accordance with the result of CREB phosphorylation, C/EBPβ protein expression was markedly decreased by berberine 1 and 3 days after induction of adipocyte differentiation. On day 5 of adipocyte differentiation, berberine still exerted an inhibitory effect on C/EBPβ protein expression (Fig 4A), but not CREB phosphorylation (Fig 4A). Berberine treatment decreased C/EBPβ protein expression at 6 h after MDI induction (Fig 4B), which was later than the inhibition of CREB phosphorylation at 2 h. However, both CREB phosphorylation and C/EBPβ protein expression were maximally suppressed by berberine on day 1 (Fig 4A).

### Berberine suppresses cAMP raising agents-induced CREB phosphorylation and C/EBPβ expression

**Fig 5:**
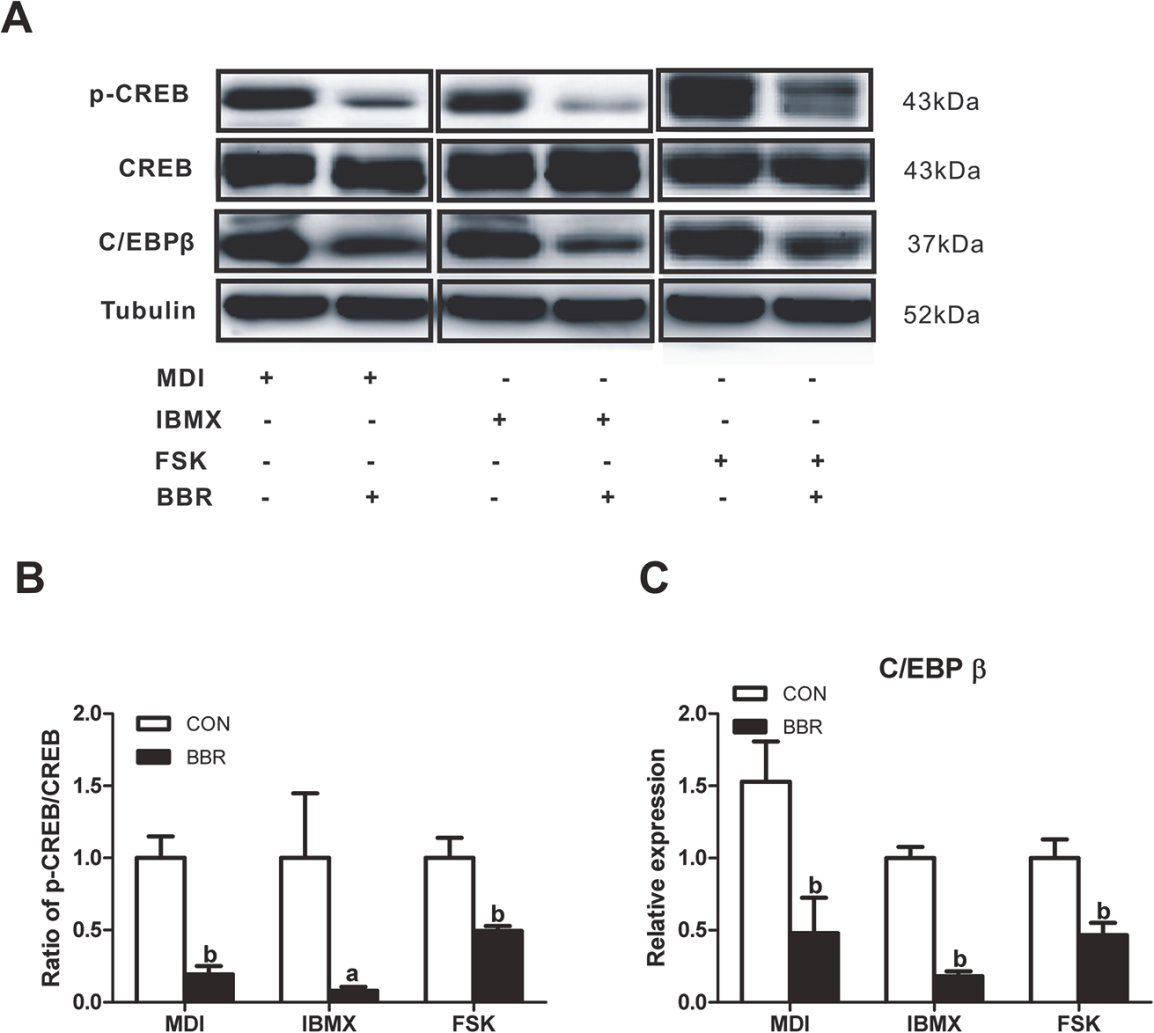
Berberine attenuates IBMX- and FSK-induced CREB phosphorylation and C/EBPβ expression. (A)3T3-L1 preadipocytes were stimulated with MDI, 0.5 mM 3-isobutyl-1-methylxanthine (IBMX) or 10 μM forskolin (FSK) for 12 h in the presence or absence of 5 μM berberine (BBR). Cell lysates were prepared for assaying CREB phosphorylation and C/EBPβ expression with Western blot. (B) The scanned bar graph shows relative folds of phosphorylated CREB over CREB. (C) The scanned bar graphs show relative C/EBPβ protein expression. Data are represented as the mean ± SD (*n* = 3). ^a^ *P*<0.05, ^b^ *P*<0.01 compared with control (CON).

It has been demonstrated that IBMX and cAMP analogues promote adipocyte differentiation [24]. We investigated whether berberine was able to inhibit CREB phosphorylation and C/EBPβ expression induced by cAMP raising agents. As shown in Fig 5A–5C, IBMX- and forskolin-stimulated CREB phosphorylations were also suppressed by berberine, along with decreased C/EBPβ protein expression. These results demonstrate that berberine inhibits CREB phosphorylation via cAMP dependent pathway.

### Impact of berberine on CRE activity

**Fig 6:**
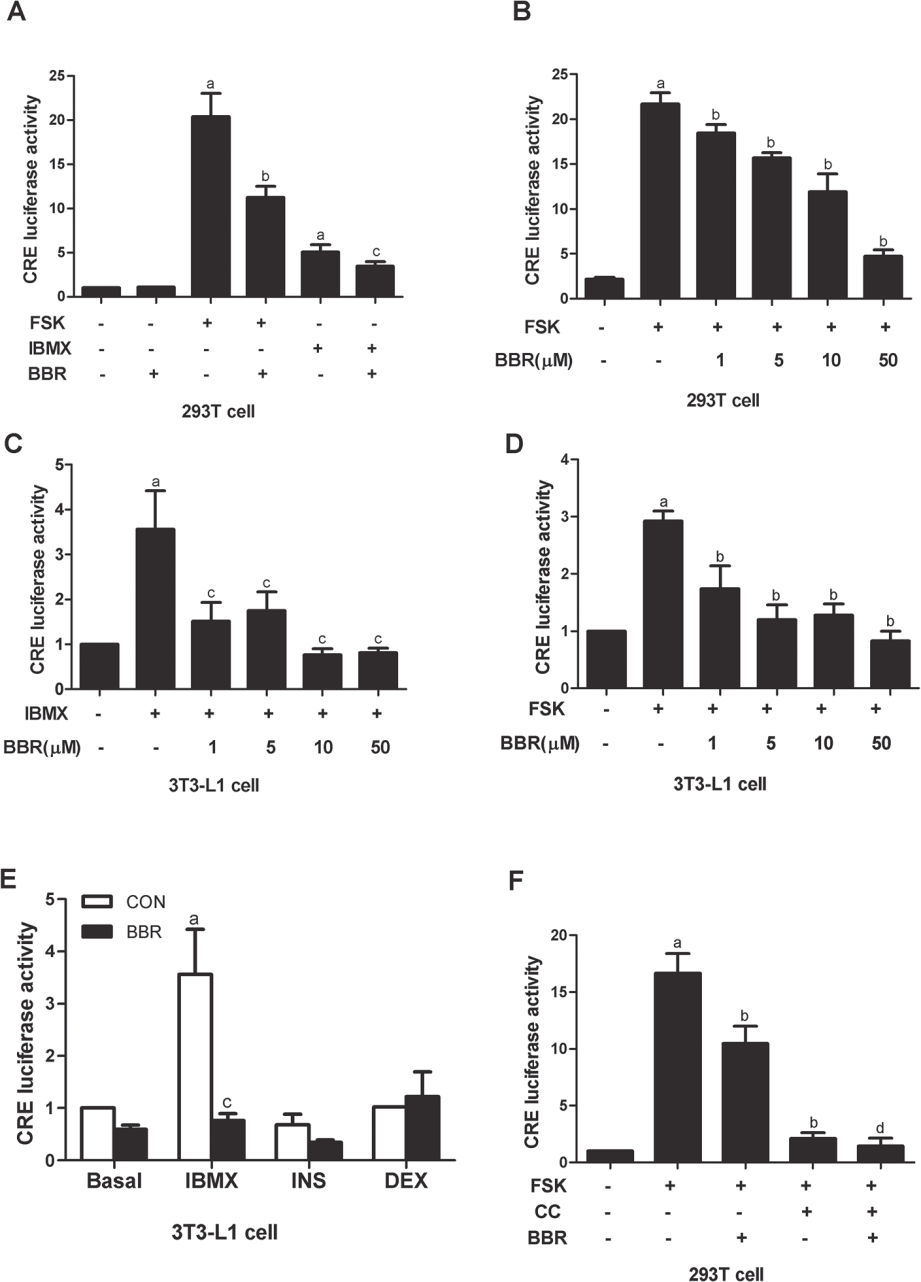
Berberine decreases IBMX- and FSK-induced CRE luciferase activity. (A and B) 293T cells transfected with CRE reporter plasmid were stimulated with 0.5 mM 3-isobutyl-1-methylxanthine (IBMX) and 10 μM forskolin (FSK) in the presence or absence of 10 μM berberine for 6 h. The dual-luciferase assay was performed. (C and D) 3T3-L1 cells transfected with CRE reporter plasmid were stimulated with 0.5 mM IBMX and 10 μM FSK in the presence of the indicated concentrations of berberine for 12 h. The dual-luciferase assay was performed. (E) 100 nM insulin and 0.25 μM dexamethasone did not stimulate CRE activity in 3T3-L1 cells. (F)10 μM Compound C (CC) suppressed forskolin-stimulated CRE activity in 293T cells. Values represent mean ± SD from at least three independent experiments. ^a^ *P*<0.05 compared with control (CON); ^b^ *P*<0.05 compared with FSK; ^c^ *P*<0.05 compared with IBMX; ^d^ *P*<0.05 compared with FSK plus CC.

CREB binds to the putative CRE in the promoters of several adipocyte-specific genes and regulates their transcription [25–27]. To functionally determine whether the berberine-induced decrease in lipid accumulation is involved in CRE activity, dual luciferase reporter assay was preformed in 293T cells and 3T3-L1 preadipocytes. Berberine treatment reduced forskolin- and IBMX-stimulated CRE luciferase activities in 293T cells (Fig 6A and 6B). Berberine exerted a similar effect in a dose-dependent manner in 3T3-L1 preadipocytes (Fig 6C and 6D). However, insulin and dexamethasone did not cause any significant increase in CRE activity (Fig 6E). Thus, MDI-induced CRE activity is mainly attributed to IBMX stimulation. In addition, compound C did not reverse berberine-suppressed CRE activity. On the contrary, it decreased forskolin-stimulated CRE activity (Fig 6F), which is in consistent with its inhibitory effect on adipocyte differentiation.

### Berberine suppresses the binding of phosphorylated CREB with the promoter of C/EBPβ

**Fig 7:**
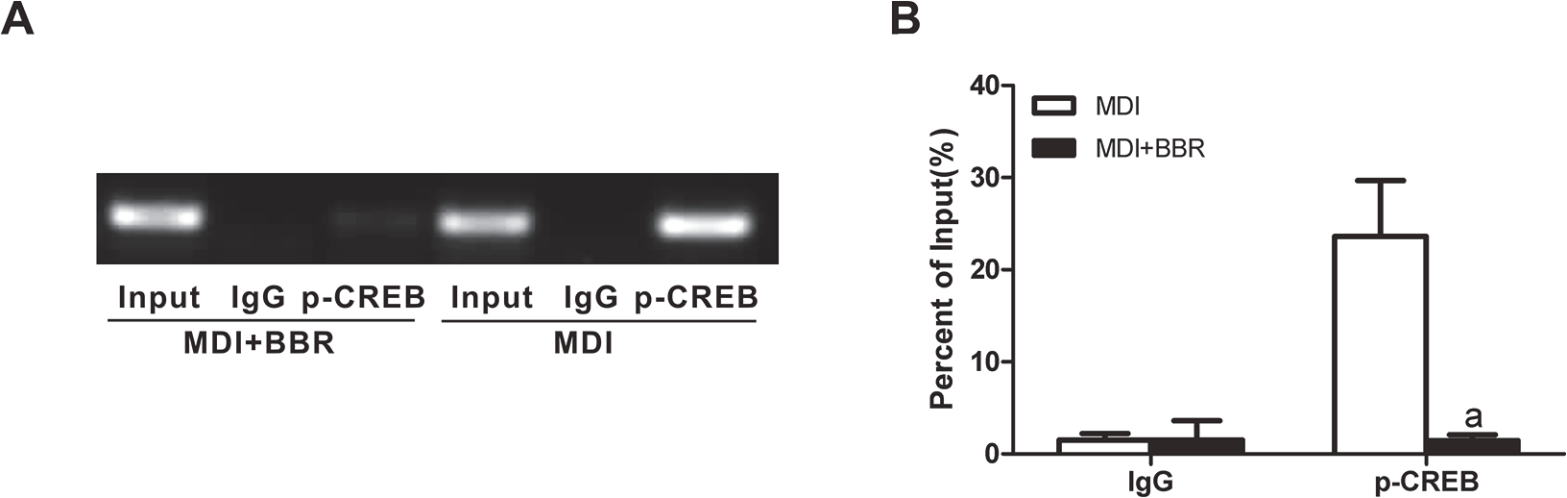
Berberine inhibits the binding of phosphorylated CREB with the promoter of C/EBPβ. (A)3T3-L1 preadipocytes were treated with MDI for 24 h in the presence or absence of 5 μM berberine, and then were cross-lined with 1% formaldehyde. Chromatin-associated DNA was fragmented and immunoprecipitated with preimmune rabbit IgG or antibodies against phosphorylated CREB. Immunoprecipitated DNA was subjected to PCR amplification with specific primers flanking the CRE sites in the promoter region (-121 to +31). (B) The relative binding level is indicated as the percentage of input DNA. The data are from three separate experiments. ^a^ *P*<0.01 compared with MDI.

The proximal promoter of the C/EBPβ gene possesses dual cis regulatory elements, both of which contain core CREB binding sites. Activated CREB binds to the dual CRE-like elements in the promoter of the C/EBPβ gene [22]. To further determine whether berberine inhibits C/EBPβ expression via CREB, we investigated the effect of berberine on the interaction of CREB with the promoter of C/EBPβ using ChIP assay. As expected, berberine abrogated the binding of phosphorylated CREB with the promoter of C/EBPβ 24 h after MDI stimulation (Fig 7A and 7B).

## Discussion

Obesity is a major risk factor for metabolic syndrome and type 2 diabetes. However, most antidiabetic drugs that are hypoglycemic also promote weight gain [20]. Therefore, it is highly desirable to develop pharmaceutical treatments for obese patients with type 2 diabetes mellitus that reduce both blood glucose level and weight. Previous clinical and experimental studies revealed the dual features of berberine. In a randomized, double-blind, and placebo-controlled trial, berberine decreased fasting and postprandial plasma glucose with a slight decrease in body weight in type 2 diabetics after 3 months treatment [6]. In db/db mice and high-fat-fed Wistar rats, berberine reduced body weight and caused a significant improvement in glucose tolerance without altering food intake. Berberine down-regulated the expression of genes involved in lipogenesis and up-regulated those involved in energy expenditure in adipose tissue [7]. In our previous study, berberine treatment for six weeks in high-fat-fed rats significantly decreased plasma glucose and body weight [8]. Adipogenesis is a major mechanism leading to weight gain and obesity. Apparently, the anti-obesity effect of berberine is mainly attributed to its inhibition of adipocyte differentiation as previously reported [9–11]. In the present study, we characterized the effect of berberine on adipocyte differentiation. Berberine reduced the accumulation of intracellular lipid in differentiated 3T3-L1 adipocytes in a dose dependent manner.

AMPK plays a key role in the regulation of carbohydrate and fat metabolism, and is a potential drug target for the treatment of obesity, diabetes, and fatty liver disease [28]. Activated AMPK causes inhibition of fatty acid synthesis through its ability to phosphorylate ACC and to down-regulate lipogenic enzyme gene transcription [29–31]. Many natural agents have been shown to activate AMPK and inhibit adipocyte differentiation as its agonist 5-aminoimidazole-4-car-boxamide riboside (AICAR) [32–34]. However, the differentiation of adipocytes was also inhibited by compound C, a specific AMPK inhibitor [21, 35]. Similar results were observed in our study (data not shown). Furthermore, compound C suppressed forskolin-stimulated CRE activity. In addition, though thiazolidinediones stimulate AMPK activation, these agents promote adipocyte differentiation via activating PPARγ [36]. Therefore, the role of AMPK in adipocyte differentiation need to be further investigated. In this current study, berberine stimulated AMPK activity throughout the course of adipogenesis process as expected (Fig 2F). But our previous studies revealed that berberine inhibited lipolysis and insulin secretion via cAMP/PKA pathway independent of AMPK [8, 15]. It has been demonstrated that cAMP/PKA pathway plays a crucial role in adipogenesis [5]. Thus, it is reasonable to deduce that berberine inhibits adipocyte differentiation via this pathway.

Cyclic AMP-dependent processes are pivotal during the early stages of adipocyte differentiation [5]. CREB was initially characterized as a cAMP target whose transcriptional activity was stimulated by cAMP-dependent PKA-catalyzed phosphorylation on serine 133. Activated CREB increases the expression of C/EBPβ at an early time point in the adipogenic differentiation process, which triggers a cascade of transcriptional events and consequently promotes activation of the C/EBPα and PPARγ2 genes. Then, C/EBPα and PPARγ2 enhance the transcription of the set of genes that give rise to the adipocyte phenotype [22]. We treated 3T3-L1 preadipocytes with berberine at the different stages of differentiation and found that berberine inhibited the initiation of adipocyte differentiation at the early stage. This result suggests that berberine may inhibit adipogenesis via cAMP/PKA pathway.

Among adipogenic inducers, IBMX and insulin have been reported to increase CREB activity [5,37]. Previously, we found that berberine stimulated glucose transport independent of insulin signal pathway while inhibited IBMX-stimulated lipolysis via increasing phosphodiesterase activity and decreasing intracellular cAMP level and hormone-sensitive lipase activity in 3T3-L1 adipocytes [12, 20]. In MIN6 cells and isolated rat islets, berberine also reduced IBMX-stimulated insulin secretion [8]. Therefore, it was likely that IBMX-potentiated CREB activity was suppressed by berberine during MDI induction of adipocyte differentiation. Sure enough, IBMX- and forskolin-stimulated CREB phosphorylations were attenuated by berberine. Our results suggest that cAMP-triggered differentiation process is the main target of berberine in suppressing adipogenesis.

As a key transcript factor, activated CREB triggers the expressions of adipogenic genes via binding to the CRE in their promoter regions [25]. Our study showed that CRE activity was stimulated by IBMX and forskolin in 293T cells and 3T3-L1 preadipocytes, but not by insulin and dexamethasone. Berberine treatment decreased IBMX- and forskolin-stimulated CRE activities. Among the downstream targets of CREB, C/EBPβ is one of the critical transcription factors involved in the initiation of mitotic clonal expansion and the induction of the expression of pleiotropic transcription factors [22]. In this study, C/EBPβ protein expression was inhibited by berberine from 6 h to 5 days after hormonal stimulation, which was later than the inhibition of CREB phosphorylation (from 2 h to 3 days after hormonal stimulation). Since activated CREB initiated C/EBPβ gene expression via binding to the dual CRE-like elements in its proximal promoter, we detected the binding of phosphorylated CREB with the promoter of C/EBPβ using ChIP assay. After MDI induction for 24 h, the binding of phosphorylated CREB with the promoter of C/EBPβ was abrogated by berberine.

There are conflicting results about whether CREB directly regulates C/EBPα and PPARγ expressions. Reusch et al reported that recombinant CREB was unable to bind to putative CRE sequences in the promoters of C/EBPα and PPARγ [27]. However, Fox et al demonstrated that CREB directly promotes PPARγ2 gene transcription [22]. It is widely recognized that both of the C/EBPα and PPARγ genes possess *cis*-C/EBP regulatory elements in their proximal promoters at which C/EBPβ binds and coordinately activates transcription [22,38]. Therefore, it is possible that berberine decreases C/EBPα and PPARγ2 expression mainly via inhibiting C/EBPβ activity. Meanwhile, we noticed that it was more obvious for berberine inhibiting the expressions of adipogenic genes such as C/EBPα, PPARγ2, SREBP1c, and LPL with the maturity of 3T3-L1 adipocyte differentiation (Fig 2A–2D). 3T3-L1 adipocytes exposed to berberine on 0–7 days accumulated less lipid droplets than those on 0–2 days (Fig 3B–3D). These results suggest that AMPK may play a role in berberine-suppressed adipogenesis via decreasing lipogenic gene expressions at the terminal differentiation stage.

In conclusion, berberine blocks the process of adipocyte differentiation mainly via suppressing CREB activity induced by cAMP elevating agents, which leads to a decrease in C/EBPβ-triggered transcriptional cascades. Our previous studies showed that berberine decreased lipolysis and insulin secretion by attenuating cAMP/PAK signaling pathway. These results suggest that the inhibition of cAMP/PKA-mediated CREB pathway mainly contributes to the anti-obesity effect of berberine. Therefore, more attention should be paid to the role of this pathway in berberine-improved metabolic effects.
